# Interleukin‐38 protects against sepsis by augmenting immunosuppressive activity of CD4^+^CD25^+^ regulatory T cells

**DOI:** 10.1111/jcmm.14902

**Published:** 2019-12-27

**Authors:** Yun Ge, Man Huang, Yao Wu, Ning Dong, Yong‐Ming Yao

**Affiliations:** ^1^ Department of General Intensive Care Unit The Second Affiliated Hospital of Zhejiang University School of Medicine Hangzhou China; ^2^ Trauma Research Center Fourth Medical Center of The Chinese PLA General Hospital Beijing China; ^3^ State Key Laboratory of Kidney Disease The Chinese PLA General Hospital Beijing China

**Keywords:** immune response, interleukin‐38, regulatory T cells, sepsis

## Abstract

Naturally occurring CD4^+^CD25^+^ regulatory T cells (Tregs) are required to limit immune‐induced pathology and to maintain homeostasis during the early‐phase of sepsis. This study aimed to investigate the role of interleukin (IL)‐38, a newly described member of the IL‐1 cytokine family, in mediated immune response of CD4^+^CD25^+^ Tregs in sepsis. Here, we provide evidence that expressions of IL‐38 and its receptor were detected in murine CD4^+^CD25^+^ Tregs. Stimulation of CD4^+^CD25^+^ Tregs with LPS markedly up‐regulated the expression of IL‐38. Treatment with rmIL‐38 dramatically enhanced the immunosuppressive activity of CD4^+^CD25^+^ Tregs after LPS stimulation and in septic mice induced by CLP, resulting in amplification of helper T cell (Th) 2 response and reduction in the proliferation of effector T cells. These effects were robustly abrogated when anti–IL‐38 antibody was administered. Administration of rmIL‐38 improved the survival rate of CLP mice. In addition, CD4^+^CD25^+^ Tregs depletion before the onset of sepsis obviously abolished IL‐38–mediated protective response. These findings suggest that IL‐38 enhances the immunosuppressive activity of CD4^+^CD25^+^ Tregs, which might contribute to the improvement of host immune function and prognosis in the setting of sepsis.

## 1 INTRODUCTION

CD4^+^CD25^+^ regulatory T cells (Tregs) can regulate host responses to infections and tumours, and contribute to the prevention of allergies, autoimmune diseases and transplant rejection.^1^, ^2^, ^3^, ^4^ CD4^+^CD25^+^ Tregs are essential for maintenance of immune homeostasis and self‐tolerance. They express several crucial molecular markers, including cytotoxic T lymphocyte–associated antigen 4 (CTLA‐4, also termed CD152), glucocorticoid‐induced tumour necrosis factor receptor (GITR) and forkhead/winged helix transcription factor p3 (Foxp3). Knockdown of these factors leads to lethal lymphoproliferative phenotypes.^5^, ^6^, ^7^, ^8^ Concomitantly, CD4^+^CD25^+^Tregs can produce various potent anti‐inflammatory cytokines, such as interleukin (IL)‐10 and transforming growth factor (TGF)‐β, which cause immunosuppression of effector T cells.^9^, ^10^, ^11^, ^12^ Moreover, CD4^+^CD25^+^Tregs can reduce IL‐2 production and inhibit polyclonal T cell activity in vitro,^13^ and they exploit distinct mechanisms to modulate responses mediated by helper T cell (Th)1, Th2 or Th17 cells.^14^, ^15^, ^16^

Sepsis is a major cause of admission to intensive care units and is associated with elevated mortality. In the development of sepsis, infection causes aberrant inflammatory and immune responses, leading to multiple organ failure.^17^, ^18^, ^19^, ^20^, ^21^ CD4^+^CD25^+^Tregs are critically participated in the maintenance of immune homeostasis in the setting of sepsis. Following recovery from severe sepsis, mice models showed increases in CD4^+^CD25^+^Tregs. In contrast, depletion of Tregs was associated with increased IL‐6 levels and mortality in the early‐phase of sepsis.^22^, ^23^, ^24^ Genetic or immunological inhibition of Tregs resulted in acute death in lipopolysaccharide (LPS)‐challenged mice because of exaggerated inflammatory response.^25^, ^26^, ^27^ Adoptive transfer of in vitro‐stimulated CD4^+^CD25^+^Tregs before or 6 hours after caecal ligation and puncture (CLP) reduced the mortality in mice and improved bacterial clearance.^28^, ^29^, ^30^ Overall, these findings indicate that CD4^+^CD25^+^ Tregs can help to limit uncontrolled inflammation, promote bacterial clearance and improve survival in the early hyper‐inflammatory stage of sepsis.

IL‐38, previously termed IL‐1F10, is a poorly characterized cytokine belonging to the IL‐1 family. IL‐38 is extensively expressed in several tissues including spleen, thymus, foetal liver, placenta, lung and heart. It is also expressed in proliferating B cells and epithelia.^31^ Interestingly, IL‐38 can be released from apoptotic cells to control inflammatory macrophage and Th17 response.^32^ IL‐38 binds to the IL‐36 receptor (IL‐36R) and has been proposed as an IL‐36R antagonist based on sequence homology studies.^33^ The expression of IL‐38 is correlated tightly with various inflammatory and autoimmune disorders such as infection, asthma, systemic lupus erythematosus, psoriatic arthritis and rheumatoid arthritis.^34^, ^35^, ^36^, ^37^, ^38^, ^39^, ^40^, ^41^, ^42^, ^43^ It was reported that IL‐38 levels were elevated in patients with sepsis, and administration of recombinant murine IL‐38 (rmIL‐38) in mice could protect against challenge with LPS and sepsis induced by CLP without affecting bacterial phagocytosis.^43^ However, little is known about the influence of IL‐38 on the activity of CD4^+^CD25^+^ Tregs in sepsis‐induced immune dysfunction.

In the current study, we aimed to investigate the role of IL‐38 in mediated activity of CD4^+^CD25^+^ Tregs during sepsis in a murine model. Our results identified a novel biological effect of IL‐38 on host immune response and elucidated its potential mechanism in the pathogenesis of sepsis.

## 2 MATERIALS AND METHODS

### 2.1 Ethics statement

All experiments were performed in accordance with the guidelines of the National Institutes of Health Guide for the Care and Use of Laboratory Animals and with the approval of the Scientific Investigation Board of the Chinese PLA General Hospital (SYXK2016‐0018), Beijing, China.

### 2.2 Animals and reagents

Male BALB/c mice aged 6‐8 weeks old were purchased from the Laboratory Animal Center of the Chinese Academy of Medical Sciences (SCXK‐jing‐2014‐0004), Beijing, China. The mice were housed in cages in a temperature‐controlled room under a 12‐hour light/dark cycle for at least 7 days before experiments.

Recombinant murine IL‐38 and anti–IL‐38 antibody were obtained from R&D Systems, Minneapolis, MN. CD4^+^CD25^+^ T cell isolation kit was purchased from MiltenyiBiotec. LPS was purchased from Sigma‐Aldrich, St. Louis, MO. Carboxyfluoresceinsuccinimidyl ester (CFSE) cell proliferation kit was obtained from Invitrogen. Enzyme‐linked immunosorbent assay (ELISA) kits for mouse IL‐10, TGF‐β1, IL‐2, IL‐4 and interferon (IFN)‐γ were obtained from ExCell Biology. Antimouse antibodies against Foxp3 [FJK‐16s, conjugated to phycoerythrin (PE)‐cyanine7], CD152 (CTLA‐4) [UC10‐4B9, conjugated to allophycocyanin (APC)], CD4 [RM4‐5, conjugated to fluorescein isothiocyanate (FITC)], CD25 (PC61.5, conjugated to PE), CD3e (clone 145‐2C11) or CD28 (clone 37.51) were obtained from eBioscience. Anti‐CD25 antibody (purified antimouse CD25 antibody, clone PC61) and rat IgG1 were purchased from Biolegend.

### 2.3 Purification and culture of CD4^+ ^CD25+Tregs and CD4^+^ CD25−T cells

CD4^+^CD25^−^ T cells and CD4^+^CD25^+^ Tregs were isolated from murine splenocytes in a two‐step procedure following the manufacturer's instructions (MiltenyiBiotec). Firstly, non‐CD4^+^ T cells were magnetically labelled with a cocktail of biotin‐conjugated antibodies (10 μL per 10^7^ cells) at 4°C for 10 minutes, followed by incubation with anti‐biotin monoclonal antibodies conjugated to microbeads (20 μL per 10^7^ cells) and CD25‐PE (10 μL per 10^7^ cells) at 4°C for 15 minutes in the dark. The cell suspensions flowed through a LD magnetic‐activated cell sorting (MACS) column to bind labelled cells and allow CD4^+^ T cells to pass through. Secondly, the flow‐through of CD4^+^ T cells was centrifuged and then incubated with anti‐PE microbeads (10 μL per 10^7^ cells) at 4°C for 15 minutes in the dark. Finally, CD4^+^CD25^+^ Tregs were retained on the MS MACS column and isolated from the pre‐enriched CD4^+^ T cell fraction. The flow‐through CD4^+^CD25^−^ T cells were collected. Cells were stained with trypan blue for counting in a haemocytometer.

CD4^+^CD25^+^Tregs and CD4^+^CD25^−^ T cells were cultured separately in RPMI 1640 medium containing 10% foetal bovine serum (FBS) in 5% CO_2_ at 37°C. Cells were treated with 2 μg/mL soluble anti‐CD3 monoclonal antibody and 2 μg/mL soluble anti‐CD28 monoclonal antibody. In some experiments, cells were also stimulated with LPS (5 μg/mL). After 2 hours, IL‐38 was added to the medium at concentrations of 50, 100 or 200 ng/mL, and cultures were incubated for 48 hours. These CD4^+^CD25^+^Treg cultures were then used for suppressive activity assays and molecular marker measurements. Culture supernatants were harvested for the evaluation of cytokines (IL‐10 and TGF‐β1).

### 2.4 Carboxyfluorescein succinimidyl ester staining

Sorted CD4^+^CD25^−^ T cells were washed with phosphate‐buffered saline (PBS) and then resuspended (10^6^/mL) in PBS containing 1 μL of CellTrace™ stock solution. The labelled cells were incubated for 20 minutes at 37°C, protected from light. Cells were pelleted by centrifugation at 400  *g* for 7 minutes and resuspended in fresh pre‐warmed complete culture medium. Cells were incubated for at least 10 minutes at room temperature to allow the CellTrace™ reagent to undergo acetate hydrolysis.

### 2.5 Suppressive activity assay

Following stimulation with LPS and IL‐38, CD4^+^CD25^+^Tregs (10^5^) were co‐cultured 1:1 with CD4^+^CD25^−^ T cells in U‐bottom 96‐well plates. Cell co‐cultures were suspended in fresh culture medium, treated with 2 μg/mL soluble anti‐CD3 monoclonal antibody and 2 μg/mL soluble anti‐CD28 antibody, incubated for 72 hours and analysed using flow cytometry. The proliferation of CFSE‐labelled CD4^+^CD25^−^ T cells was determined using a FlowJo system (BD Bioscience). Cell supernatants were harvested for ELISA (IL‐2, IL‐4 and IFN‐γ).

### 2.6 Flow cytometry

CD4^+^CD25^+^ Tregs were collected, washed twice with PBS, incubated in fixation/permeabilization solution, washed with permeabilization buffer and incubated for 30 minutes (at 4°C in the dark) with PE‐cyanine7‐conjugated antibody against Foxp3 or antimouse APC‐conjugated antibody against CD152 (CTLA‐4). Then, cells were washed with PBS and fixed in 1% formaldehyde solution for flow cytometry analysis performed with a FACSCalibur (BD Bioscience).

### 2.7 ELISA

Levels of IL‐2, IL‐4, IL‐10, IFN‐γ and TGF‐β1 were analysed by ELISA following manufacturer's instructions. Chromogenic reactions were terminated by adding 100 μL of orthophosphoric acid. Plates were measured on a microplate reader (Spectra MR; Dynex).

### 2.8 Laser scanning confocal microscopy

Cultured murine CD4^+^CD25^+^ Tregs (10^6^) were collected, washed twice with PBS and suspended in 500 μL of PBS. Then, samples were incubated with polyclonal rabbit anti–IL‐38 antibody (1:500), followed by FITC‐conjugated AffiniPure goat anti‐rabbit IgG (1:100) for 1 hour at 37°C. Finally, cells were stained with 4′,6‐diamidino‐2‐phenylindole (DAPI) and imaged using a laser scanning confocal microscope (Leica).

### 2.9 Quantitative reverse transcription‐polymerase chain reaction (PCR)

Total mRNA from murine CD4^+^CD25^+^ Tregs was extracted using Trizol reagent (Invitrogen) and RNeasyMini kit (Qiagen), and then reverse‐transcribed using an iScript™ kit (Bio‐Rad) according to the manufacturers' instructions. Quantitative PCR was performed using a CFX96TM real‐time PCR Detection System and SYBR Green Master Mix (Bio‐Rad). β‐actin was used as an endogenous control. The amplification efficiency was 0.90‐0.99. Data were analysed using the comparative cycle threshold (Ct) method.

### 2.10 Western blotting

IL‐38 expression in murine CD4^+^CD25^+^ Tregs was measured by Western blot following the manufacturer's instructions. In brief, nitrocellulose membranes were incubated at 4°C overnight with polyclonal rabbit antibodies against IL‐38 (1:1000) and monoclonal mouse antibody against β‐actin (1:1000), followed by incubation with horseradish peroxidase‐conjugated polyclonal goat anti‐rabbit secondary antibody (1:5000) or monoclonal rabbit antimouse antibody (1:5000) at room temperature for 1 hour. Finally, Western blot bands were visualized using ECL^Plus^ (Amersham Biosciences) on an ImageQuant LAS 4000 biomolecular imager (GE Healthcare Life Sciences) and analysed using ImageJ software (US National Institutes of Health, https://imagej.nih.gov/ij/).

### 2.11 Sepsis model

Caecal ligation and puncture was performed to establish a mouse model of polymicrobial sepsis. In brief, mice were anaesthetized intraperitoneally with 0.3% sodium pentobarbital, and the caecum was exposed, ligatured at its external third, and punctured with a 26‐gauge needle (non‐severe CLP) or a 22‐gauge needle (severe CLP). Subsequently, the caecum was returned, and the incision was closed. In the sham group, animals underwent an identical procedure without caecum ligation or puncture. Mice received 1 mL of 0.9% subcutaneous saline solution for resuscitation. At the end of the survival experiment, all living mice were anaesthesized using 0.3% sodium pentobarbital and sacrificed by cervical dislocation.

### 2.12 Effect of IL‐38 administration on sepsis in mice

For cytokine treatment, severe CLP animals were injected intraperitoneally with 1 μg of IL‐38 at 2 hours before or after severe CLP. In some experiments, severe CLP mice were administered 0.25, 0.5 or 1 μg IL‐38 per animal at 2 hours before severe CLP. Control animals received PBS vehicle in a similar fashion.

### 2.13 Effect of anti–IL‐38 antibody on sepsis in mice

Non‐severe CLP mice were injected intraperitoneally with anti–IL‐38 antibody (50 μg per mouse) immediately after non‐severe CLP, followed by a booster dose of 50 μg 24 hours later. Rat IgG1 was administered as a control.

### 2.14 Depletion of CD4^+ ^CD25^+^ Tregs by anti‐CD25 antibody

Mice were injected intraperitoneally with 200 µg of anti‐CD25 antibody (PC61) or rat IgG1 in 200 µL of PBS 48 hours before CLP. Depletion of CD4^+^CD25^+^ Tregs was analysed by flow cytometry on the basis of expression of CD4^+^CD25^+^Foxp3^+^ cells.

### 2.15 Statistical analyses

Data from measured parameters were expressed as the mean ± standard deviation (SD) of at least three experiments. Differences between groups were evaluated for significance using the Brown‐Forsythe test or, when appropriate, one‐way ANOVA followed by Dunnett's test. Survival rate was performed using Kaplan‐Meier survival curves followed by the log‐rank test. All analysis was done using SPSS (IBM). *P* < .05 was considered statistically significant.

## 3 RESULTS

### 3.1 IL‐38 expression in murine CD4^+^CD25^+^Tregs following LPS stimulation

**Figure 1:**
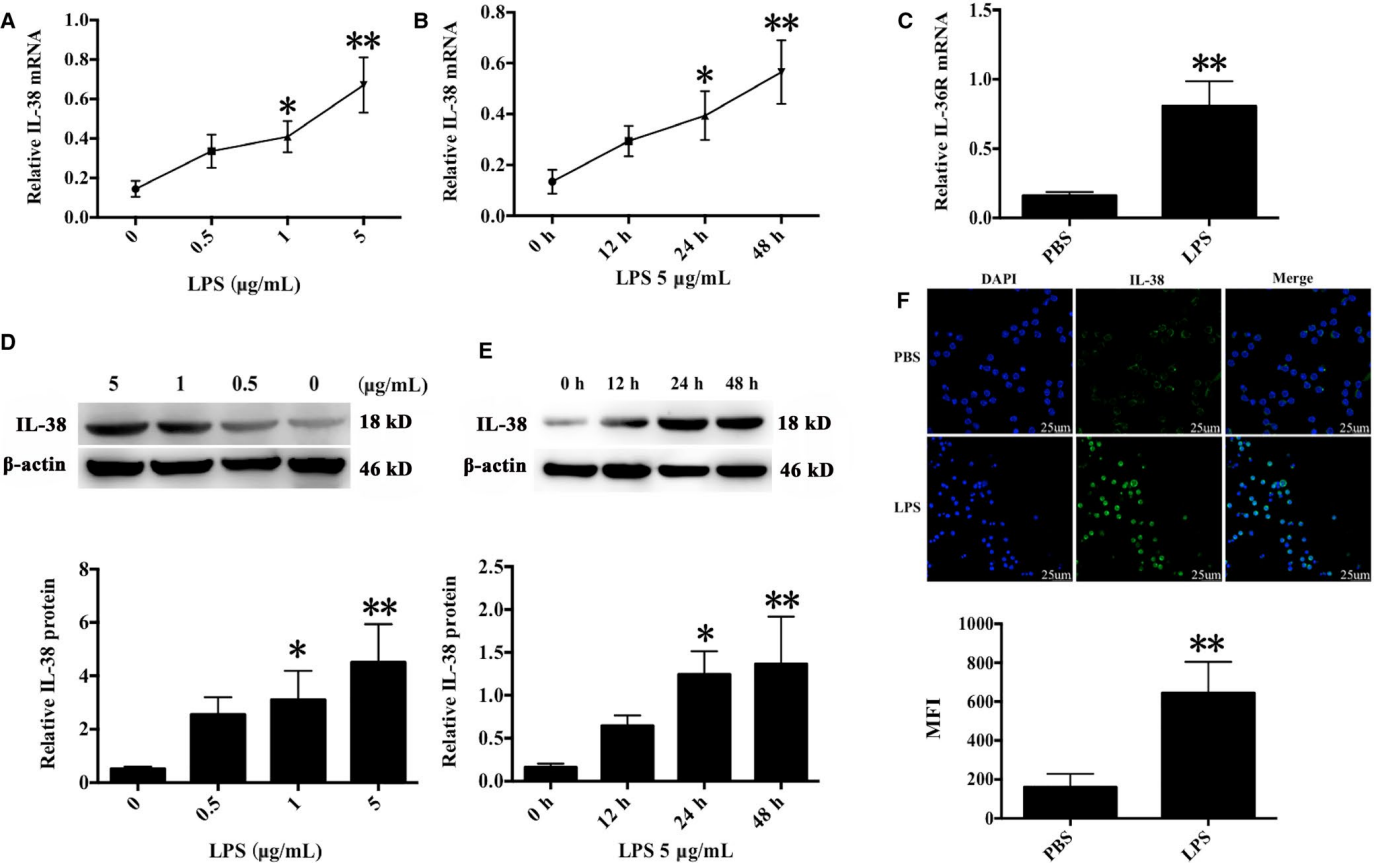
Expression of IL‐38 and its receptor IL‐36R in murine CD4^+^ CD25^+^ Tregs after LPS stimulation. CD4^+ ^CD25^+^ Tregs were isolated from murine splenocytes. (A, B) IL‐38 mRNA levels in CD4^+^ CD25+Tregs stimulated with 0.5, 1 or 5 μg/mL LPS for 12, 24 or 48 h. C, IL‐36R mRNA expression in CD4^+^ CD25+Tregs stimulated with LPS (5 μg/mL) for 48 h. (D, E) IL‐38 protein levels in CD4^+^ CD25^+^ Tregs stimulated with 0.5, 1 or 5 μg/mL LPS for 12, 24 or 48 h. F, Mean fluorescence intensity (MFI) of IL‐38 expression in CD4^+^ CD25^+^ Tregs stimulated with LPS (5 μg/mL) for 48 h, as analysed using laser scanning confocal microscopy. *P < .05, **P < .01, vs the PBS control group (n = 3 per group)

It has been documented that IL‐38 expression is presented in B cells and epithelia, as well as apoptotic cells. IL‐38 can be induced by pro‐inflammatory stimuli in various pathological states. Previous studies showed that LPS treatment promoted CD4^+^CD25^+^Tregs proliferation and enhanced their suppressive activities, providing evidence that Tregs directly responded to pro‐inflammatory bacterial products and contributed to inflammation control.^16^ In this study, IL‐38 and its receptor IL‐36R were positively expressed in murine CD4^+^CD25^+^ Tregs, and their expressions were markedly higher after stimulation with 5 μg/mL LPS for 24 and 48 hours than that of the controls treated with PBS (Figure 1).

### 3.2 Effects of IL‐38 on expression of Foxp3 and CD152 (CTLA‐4) in CD4^+^ CD25+Tregs

**Figure 2:**
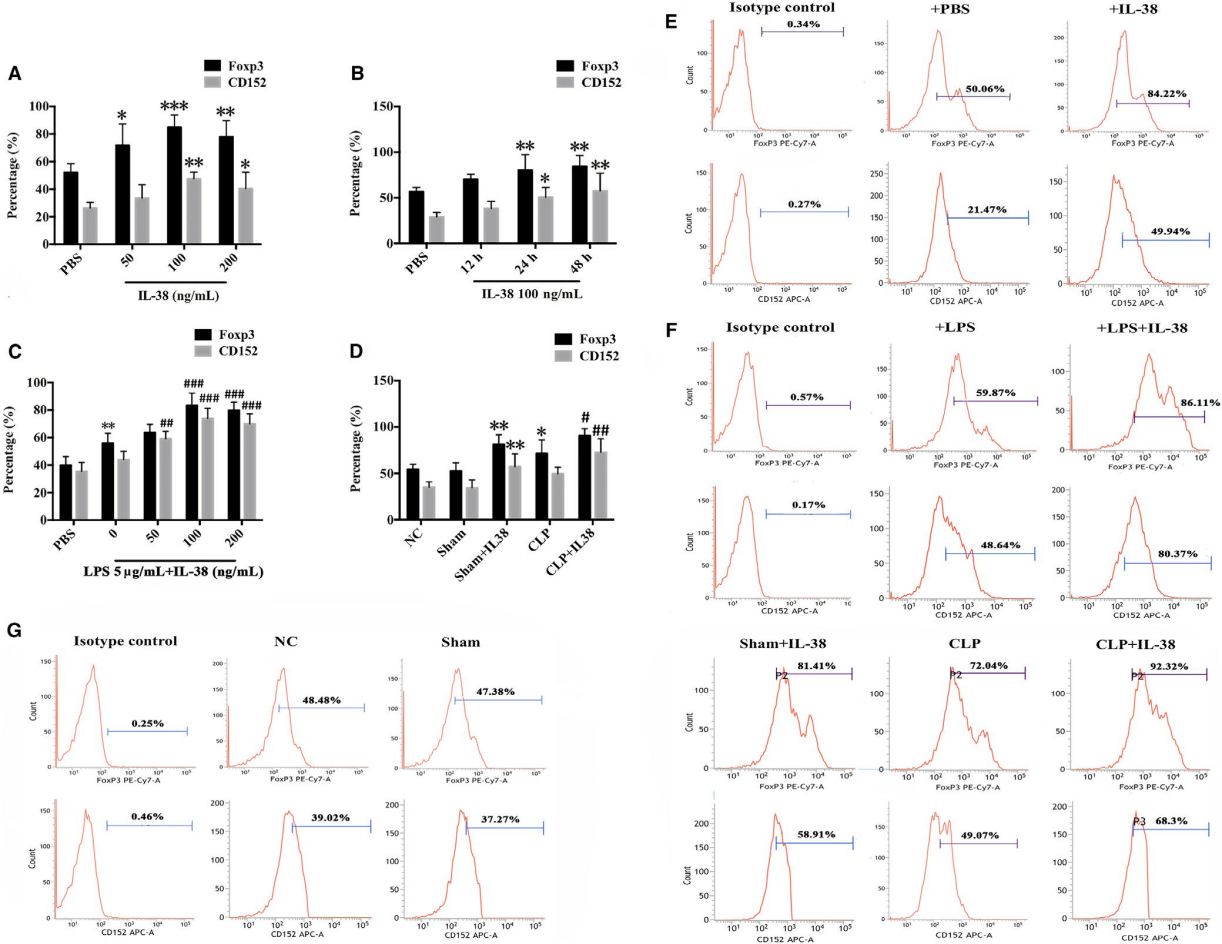
Effects of IL‐38 on Foxp3 and CD152 (CTLA‐4) expression in murine CD4^+^ CD25^+^ Tregs. CD4^+^ CD25+Tregs were isolated from murine splenocytes. (A, E) Expression of Foxp3 and CD152 in murine CD4^+ ^CD25+Tregs treated with PBS or 50, 100 or 200 ng/mL IL‐38 for 48 h. *P < .05, **P < .01, ***P < .001 vs the PBS control group. (B, E) Expression of Foxp3 and CD152 in murine CD4^+^ CD25^+^ Tregs stimulated with 100 ng/mL IL‐38 for 12, 24 or 48 h. *P < .05, **P < .01, vs the PBS control group. (C, F) Foxp3 and CD152 expression in CD4^+^ CD25^+^ Tregs incubated for 48 h with LPS (5 μg/mL) and IL‐38 (50, 100, or 200 ng/mL). **P < .01 vs the PBS control group; ^##^ *P* < .01, ^###^ *P* < .001 vs. the LPS group. (D, G) Expression of Foxp3 and CD152 in CD4^+^ CD25+Tregs isolated from the spleens of mice treated intraperitoneally with IL‐38 (1 μg per mouse) or PBS (the control group), which underwent the caecal ligation or puncture (CLP) procedure 2 h later. Mice in the sham group were subjected to the same procedure without CLP. *P < .05, **P < .01 vs the negative control (NC) group; ^#^ *P* < .05, ^##^ *P* < .01 vs the CLP group (n = 6 per group)

Effects of IL‐38 on expression of Foxp3 and CD152 were measured by flow cytometry after incubation of CD4^+^CD25^+^ Tregs. Treatment of CD4^+^CD25^+^Tregs with IL‐38 at concentrations of 100‐200 ng/mL for 24‐48 hours significantly enhanced expression of Foxp3 and CD152, while treatment with a concentration of 50 ng/mL IL‐38 showed little effect (Figure 2A,B,E). The peak expression of Foxp3 and CD152 was observed after 48 hours incubation with 100 ng/mL IL‐38. Meanwhile, LPS stimulation increased CD152 and Foxp3 expression in CD4^+^CD25^+^Tregs (Figure 2C,F), and further up‐regulation was triggered by IL‐38 at 100 or 200 ng/mL (Figure 2C,F). In addition, treatment with IL‐38 prior to CLP led to remarkably higher levels of CD152 and Foxp3 in CD4^+^CD25^+^Tregs at 48 hours after operation (Figure 2D,G).

### 3.3 Effects of IL‐38 on productions of IL‐10 and TGF‐β1 in CD4^+ ^CD25^+^ Tregs

**Figure 3:**
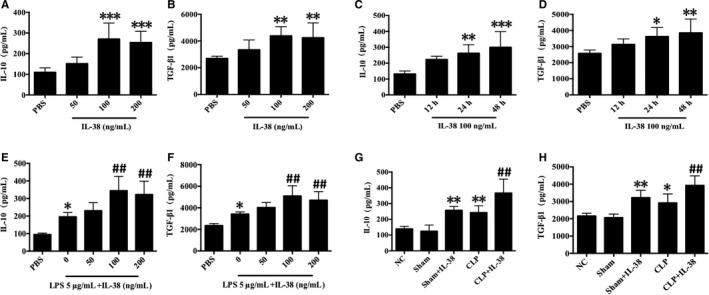
Influence of IL‐38 on the production of IL‐10 and TGF‐β1 in murine CD4^+^ CD25^+^ Tregs. CD4^+^ CD25^+^ Tregs were isolated from murine splenocytes. (A‐D) IL‐10 and TGF‐β1 levels in supernatants from murine CD4^+^ CD25^+^ Tregs treated with IL‐38 at 50, 100 or 200 ng/mL for 12, 24 or 48 h, as measured by ELISA. *P < .05, ***P < .01, ***P < .001 vs the PBS control group. (E, F) IL‐10 and TGF‐β1 levels in supernatants from CD4^+^ CD25^+^ Tregs stimulated for 48 h with LPS (5 μg/mL) together with IL‐38 at 50, 100 or 200 ng/mL, as assessed by ELISA. *P < .05 vs the PBS control group; ##*P* < .01 vs the LPS group. (G, H) Levels of IL‐10 and TGF‐β1 in mice intraperitoneally injected withIL‐38 (1 μg per mouse) or PBS (the control group), which underwent CLP 2 h later. *P < .05, **P < .01 vs the NC group; ^##^ *P* < .01 vs the CLP group (n = 6 per group)

We examined the production of anti‐inflammatory cytokines IL‐10 and TGF‐β1 in the presence of IL‐38. Treatment of CD4^+^CD25^+^Tregs with IL‐38 at 100 or 200 ng/mL induced marked elevations of TGF‐β1 and IL‐10 levels at 12, 24 and 48 hours (Figure 3A‐D). LPS stimulation markedly promoted the secretion of TGF‐β1 and IL‐10 at 48 hours, and the production of both cytokines was higher when CD4^+^CD25^+^Tregs were treated with IL‐38 and LPS (Figure 3E,F). Moreover, CLP per se up‐regulated IL‐10 and TGF‐β1 levels in mice, while higher levels of TGF‐β1 and IL‐10 were observed after IL‐38 administration in the setting of sepsis (Figure 3G,H).

### 3.4 Influence of IL‐38 on CD4^+ ^CD25^+^ Treg‐mediated immunosuppression

**Figure 4:**
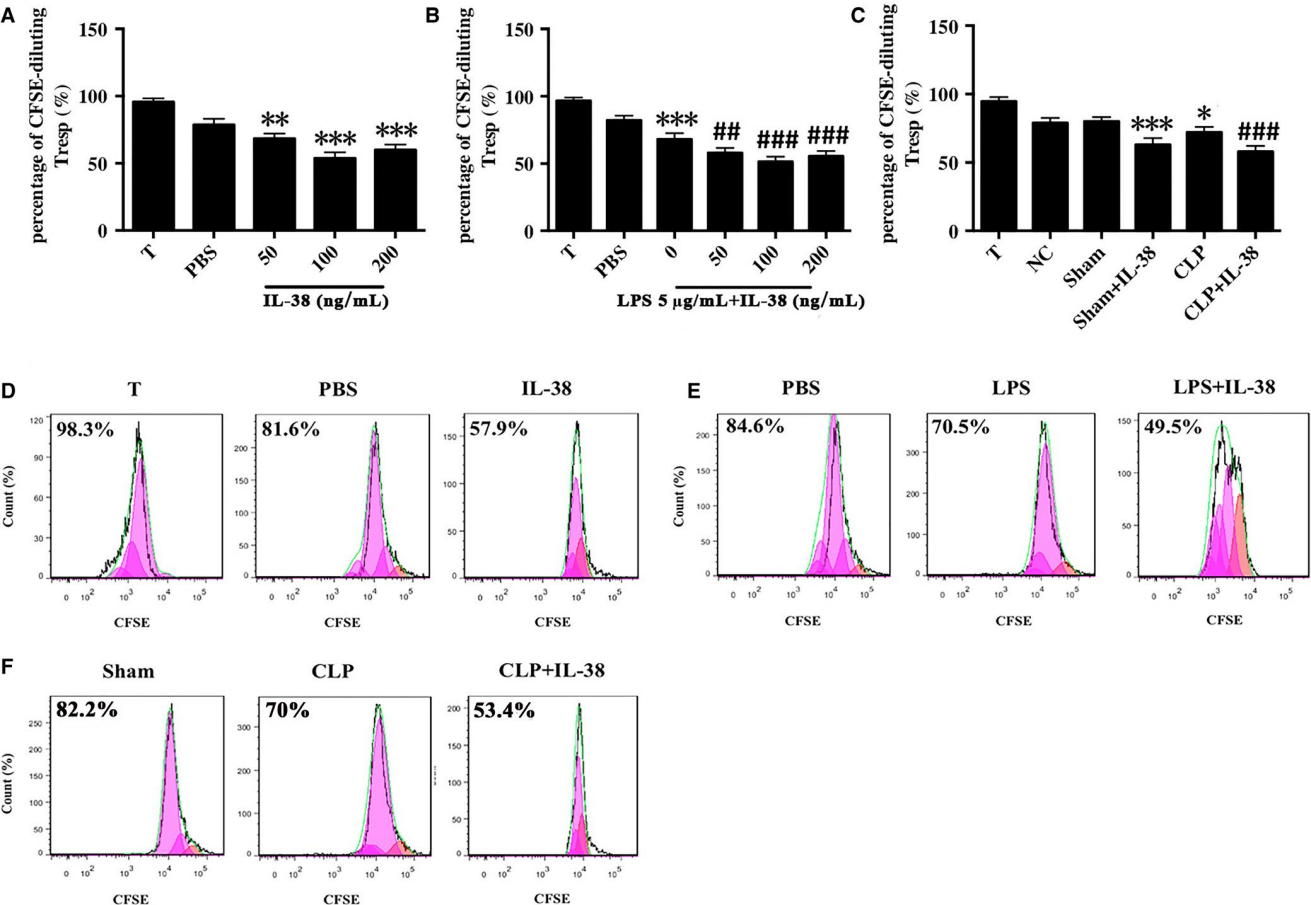
Effect of IL‐38 on the suppressive activity of murine CD4^+^ CD25^+^ Tregs isolated from murine splenocytes. CD4^+^ CD25^−^ T cells and CD4^+^ CD25^+^ Tregs were isolated from murine splenocytes. (A, B, D, E) Proliferation of CD4^+^ CD25^−^ T cells examined by CFSE staining after stimulation for 48 h with IL‐38 at 50, 100 or 200 ng/mL in the presence or absence of LPS (5 μg/mL). CD4^+^ CD25^+^ Tregs were incubated with CFSE‐labelled CD4^+^ CD25^−^ T cells in a 1:1 ratio for 72 h. **P < .01, ***P < .001 vs the PBS control group; ^##^ *P* < .01, ^###^ *P* < .001 vs the LPS group. (C, F) Proliferative activity of CD4^+^ CD25^−^ T cells examined by CFSE staining in mice that received an intra‐abdominal injection of IL‐38 (1 μg per mouse) or PBS (the control group) and that underwent CLP 2 h later. CD4^+^ CD25^+^ Tregs were isolated from the spleens of mice that underwent different treatments at 48 h and co‐cultured with CFSE‐labelled CD4^+^ CD25^−^ T cells at a 1:1 ratio for 72 h. *P < .05, ***P < .001 vs. the PBS control group; **P < .01 vs the NC group; ^###^ *P* < .001 vs the CLP group (n = 6 per group)

CD4^+^CD25^+^ Tregs were pre‐incubated with IL‐38 for 48 hours and then co‐cultured with CD4^+^CD25^−^ T cells in a 1:1 ratio. Cell co‐cultures were incubated for 72 hours, and then, CD4^+^CD25^−^ T cell proliferation was assessed by CFSE staining. Co‐culture of CD4^+^CD25^−^ T cells with CD4^+^CD25^+^Tregs pre‐treated with IL‐38 at 50, 100 or 200 ng/mL induced stronger suppression of lymphocyte proliferation than the co‐culture of CD4^+^CD25^−^ T cells with CD4^+^CD25^+^Tregs pre‐treated with PBS (Figure 4A,D). LPS stimulation strengthened the immunosuppressive ability of CD4^+^CD25^+^ Tregs, and these effects were increased when CD4^+^CD25^+^ Tregs were treated with IL‐38 (100 ng/mL) (Figure 4B,E). Consistently, CD4^+^CD25^+^ Tregs from CLP mice that had been treated preoperatively with IL‐38 obviously dampened CD4^+^CD25^−^ T cell proliferation (Figure 4C,F).

### 3.5 Impact of IL‐38 on the secretion of IL‐2, IL‐4 and IFN‐γ in CD4^+ ^CD25^−^ T cells

**Figure 5:**
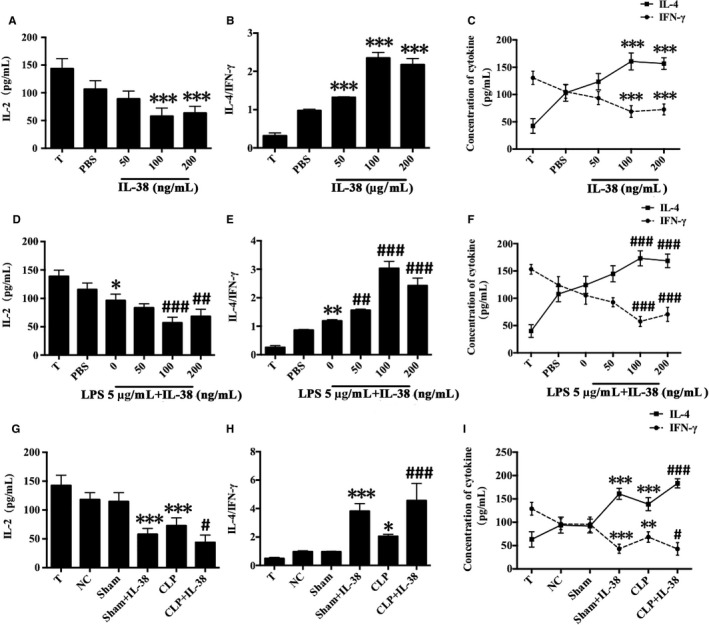
Impact of IL‐38 on IL‐2, IL‐4 and IFN‐γ levels in CD4^+^ CD25^−^ T cells in co‐culture experiments. CD4^+^ CD25^−^ T cells and CD4^+^ CD25^+^ Tregs were isolated from murine splenocytes. CD4^+^ CD25^+^ Tregs were treated with IL‐38 (50, 100 or 200 ng/mL) and then co‐cultured with CD4^+^ CD25^−^ T cells. (A‐F) IFN‐γ, IL‐2 and IL‐4 levels in the supernatants of CD4^+^ CD25^+^ Tregs co‐cultured with CD4^+^ CD25^−^ T cells after 72 h, based on ELISA. *P < .05, **P < .01, ***P < .0001 vs the PBS control group; ^##^ *P* < .01, ^###^ *P* < .001 vs the LPS group (n = 6 per group). (G‐I) IL‐2, IL‐4 and IFN‐γ levels in the supernatants of CD4^+^ CD25^+^ Tregs co‐cultured with CD4^+^ CD25^−^ T cells after 72 h. *P < .05, ***P < .01 vs the NC group; ^#^ *P* < .05, ^###^ *P* < .001 vs the CLP group (n = 6 per group)

Cytokine concentrations in the supernatants of co‐cultures of CD4^+^CD25^+^ Tregs (pre‐treated with IL‐38) and CD4^+^CD25^−^ T cells were measured at 72 hours. IL‐4 levels were significantly higher, while IL‐2 and IFN‐γ levels were lower, following IL‐38 treatment with 100 or 200 ng/mL but not 50 ng/mL (Figure 5A‐C). IL‐38 at 50, 100 or 200 ng/mL substantially elevated the ratio of IL‐4 to IFN‐γ, which suggested a substantial change in T cell polarization (Figure 5B). In addition, we found that co‐culture of CD4^+^CD25^+^Tregs with T cells in the presence of LPS showed increased levels of IL‐4 and IL‐4/IFN‐γ ratio, and reduced IL‐2 and IFN‐γ in T cells, indicating a Th2 response (Figure 5D‐F). Treatment with IL‐38 strongly amplified these responses, further promoting a Th2 response in T lymphocytes. Similar changes in IL‐2, IL‐4, IFN‐γ levels and IL‐4/IFN‐γ ratio were noted in CLP mice after IL‐38 administration (Figure 5G‐I).

### 3.6 Blockade of IL‐38 down‐regulated the capacity of IL‐38 to activate CD4^+ ^CD25^+^ Tregs

**Figure 6:**
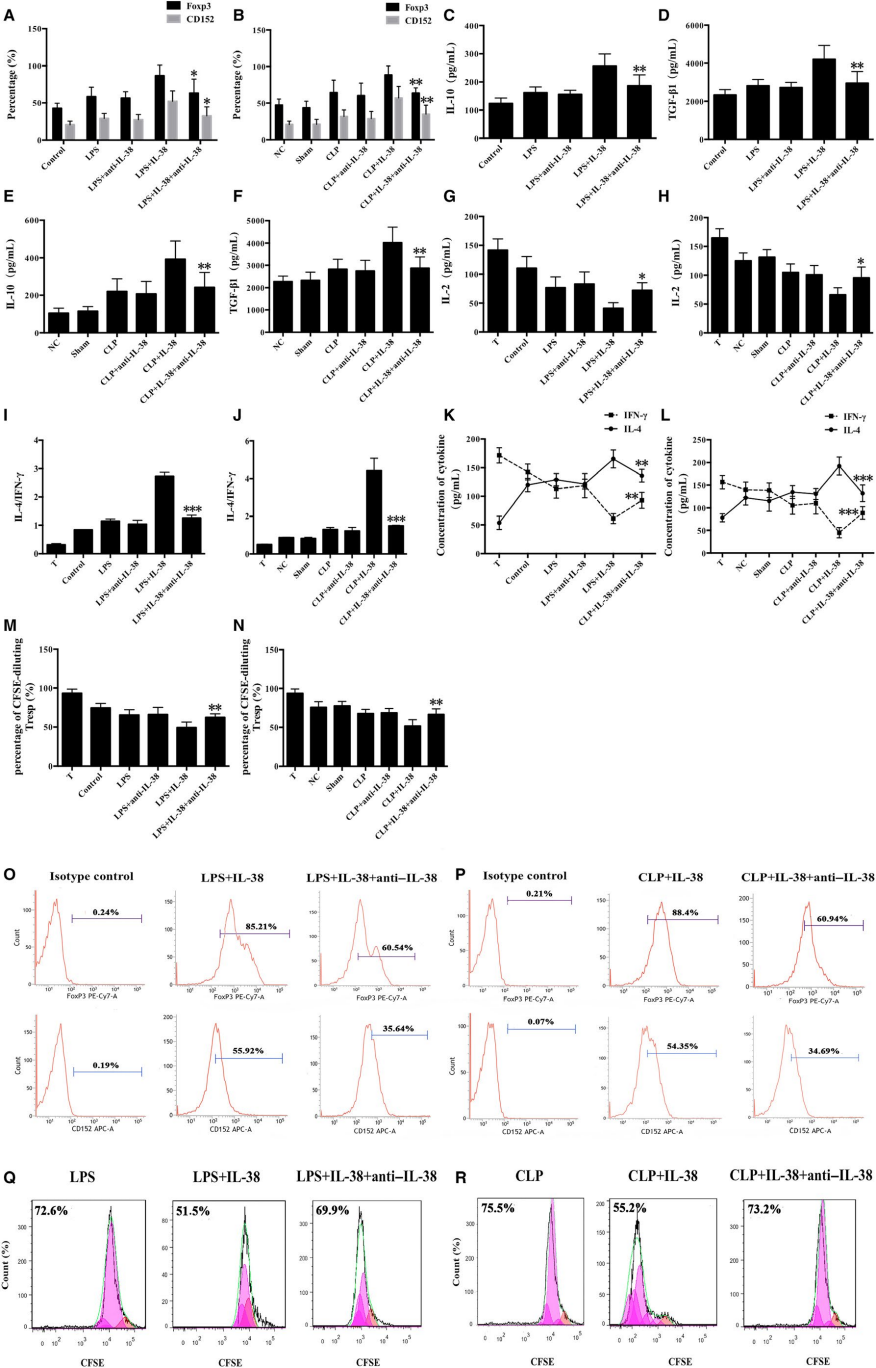
Blockade of IL‐38 down‐regulated the capacity of IL‐38 to activate CD4^+^ CD25^+^ Tregs. CD4^+^ CD25^−^ T cells and CD4^+^ CD25^+^ Tregs were isolated from murine splenocytes. After pre‐stimulation with IL‐38 at 100 ng/mL, CD4^+^ CD25^+^ Tregs were treated for 48 h with anti–IL‐38antibody (500 ng/mL) together with LPS (5 μg/mL). In other experiments, mice were intraperitoneally treated IL‐38 (1 μg per animal) then subjected to CLP 2 h later and finally given anti–IL‐38 antibody intraperitoneally (50 μg per mouse) immediately after CLP, followed 24 h later by a booster dose of 50 μg. (A, B, O, P) Expression of Foxp3 and CD152 at 48 hours performed with flow cytometry. *P < .05, **P < .01 vs the LPS + IL‐38 group or the CLP + IL‐38 group (n = 6 per group). (C‐F) Levels of IL‐10 and TGF‐β1 in the supernatants based on ELISA. **P < .01 vs the LPS + IL‐38 group or the CLP + IL‐38 group (n = 6 per group). (G‐L) IFN‐γ, IL‐2 and IL‐4 levels in the supernatants based on ELISA. ***P < .001 vs the LPS + IL‐38 group or the CLP + IL‐38 group (n = 6 per group). (M, N, Q, R) Proliferation of CD4^+^ CD25^−^ T cells according to CFSE staining after 48‐h co‐culture with CD4^+^ CD25^+^ Tregs. **P < .01 vs. the LPS + IL‐38 group or the CLP + IL‐38 group (n = 6 per group)

In order to assess the possible effect of IL‐38 on the immunosuppressive ability of CD4^+^CD25^+^ Tregs in sepsis, we stimulated cells with LPS followed by treatment with IL‐38 and anti–IL‐38 antibody. Anti–IL‐38 antibody substantially reversed IL‐38–induced effects on CD4^+^CD25^+^ Tregs, based on the measurement of CD152 and Foxp3 expression; levels of IL‐10, TGF‐β1, IL‐2, IL‐4 and IFN‐γ, and proliferative activity of CD4^+^CD25^−^ T cells co‐cultured with CD4^+^CD25^+^Tregs (Figure 6). Likely, administration of anti–IL‐38 antibody to CLP mice remarkably reduced the ability of IL‐38 to activate CD4^+^CD25^+^Tregs (Figure 6).

### 3.7 Impact of IL‐38 on the survival rate of septic mice

**Figure 7:**
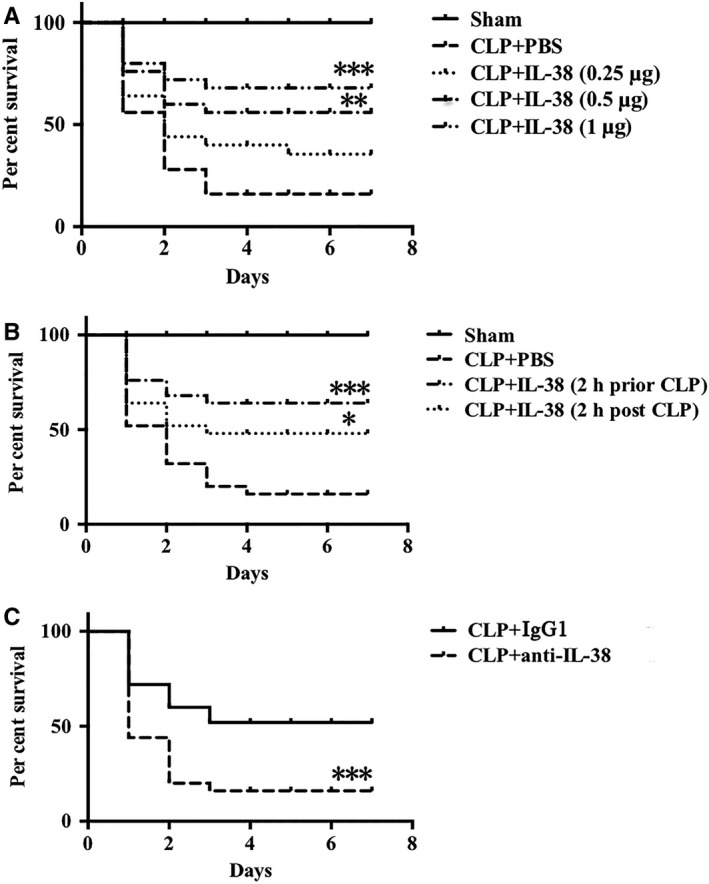
Effects of IL‐38 on the 7‐day survival rate of septic mice (A) Mice were treated with IL‐38 at 0.25, 0.5 or 1 μg per mouse and challenged with severe CLP 2 h later. In the control group, an equal volume of PBS was injected intraperitoneally. The results of each treatment group were compared with the results of the CLP + PBS group based on the log‐rank test (n = 25 per group). **P < .01, ***P < .001 vs the CLP + PBS group. B, Mice received an intra‐abdominal injection of IL‐38 (1 μg per animal) at 2 h before or after severe CLP. *P < .05, ***P < .001 vs the CLP + PBS group (n = 25 per group). C, Mice underwent non‐severe CLP and immediately injected intraperitoneally with anti–IL‐38 antibody (50 μg per mouse, followed by a booster dose of 50 μg 24 h later). Control animals received rat IgG1. ***P < .001 vs. the CLP + IgG1group

To evaluate whether the influence of IL‐38 on CD4^+^CD25^+^Tregs affected septic outcomes, we further examined the impact of IL‐38 on the mortality rate of severe CLP mice. It revealed that IL‐38 remarkably decreased CLP‐associated mortality when administered 2 hours before and after CLP (Figure 7A,B). These results showed that IL‐38, particularly when delivered early, could provide therapeutic benefits against sepsis. To investigate the potential detrimental impact of blocking IL‐38 on sepsis, we established a model of non‐severe CLP followed by anti–IL‐38 antibody administration. In these animals, CLP‐induced mortality was higher after treatment with anti–IL‐38 antibody (Figure 7C).

### 3.8 CD4^+ ^CD25^+^ Tregs were required for IL‐38–mediated protection against sepsis

**Figure 8:**
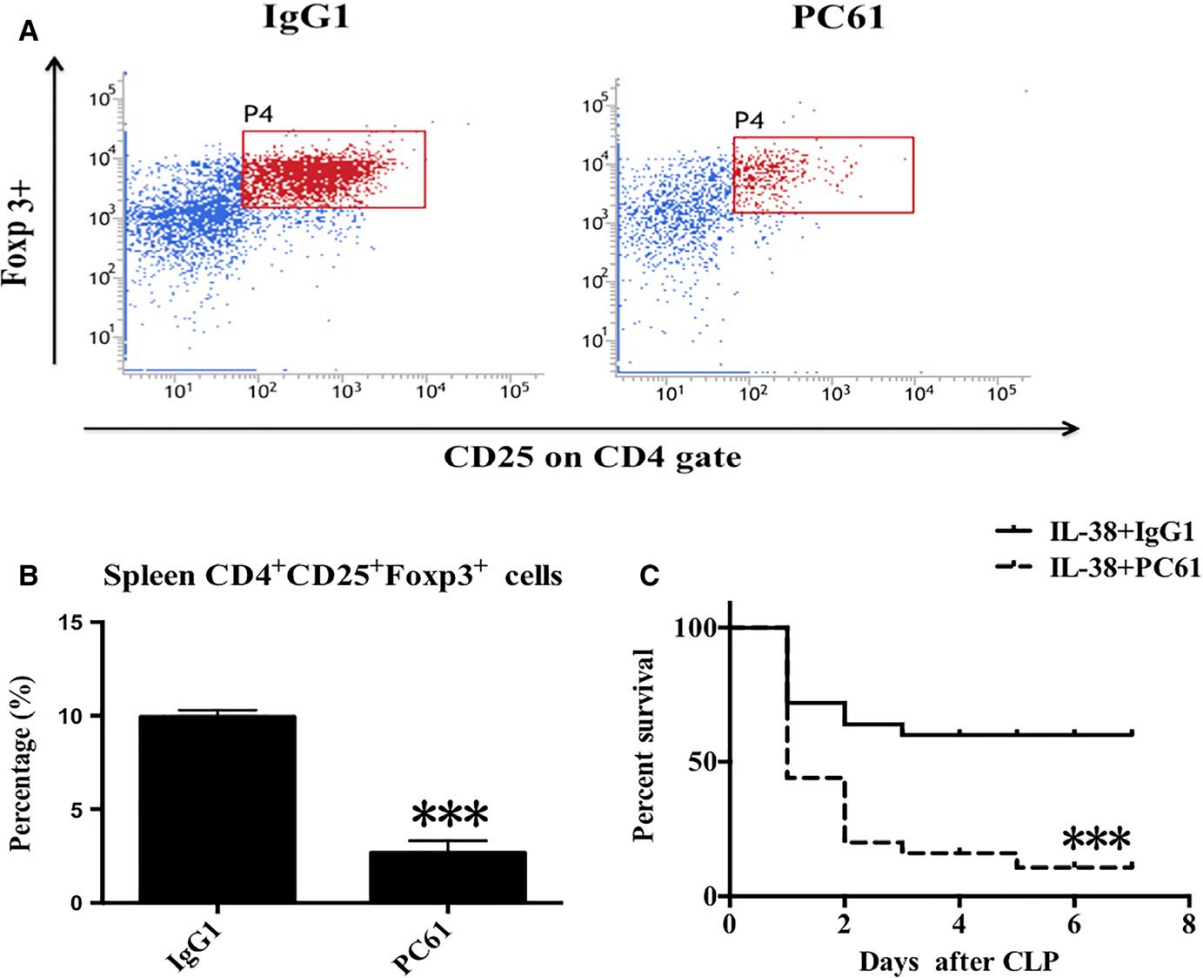
Impact of CD4^+^ CD25^+^ Treg depletion on IL‐38–mediated protection against sepsis CD4^+^ CD25^+^ Tregs was isolated from murine splenocytes. (A, B) Flow cytometry plots showed CD4^+^ CD25^+^ Tregs in the spleen at 48 h after treatment with anti‐CD25 antibody (PC61) (n = 6 per group). ***P < .001 vs mice treated with IgG1 as a control. C, Survival rates of mice depleted of CD4^+^ CD25^+^ Tregs by PC61 and treated with IL‐38 after severe CLP. Groups were compared by the log‐rank test (n = 25 per group). ***P < .001 vs mice treated with IgG1 as a control

We studied the potential role of CD4^+^CD25^+^Tregs in IL‐38–mediated protection against sepsis in vivo. Treatment with anti‐CD25 antibody greatly reduced percentages of splenic CD4^+^CD25^+^ Tregs (Figure 8A,B). Strikingly, depletion of CD4^+^CD25^+^ Tregs decreased the survival rate of septic mice treated with IL‐38 (Figure 8C), suggesting that CD4^+^CD25^+^Tregs were required for IL‐38‐induced protection following septic challenge.

## 4 DISCUSSION

There is no specific pharmacotherapy for septic patients, although manipulation of immune response may be a promising strategy for an effective treatment.^17^, ^18^, ^19^, ^20^, ^21^, ^22^, ^23^, ^24^ It is well known that Tregs play a critical role in regulating immune homeostasis, including several pathological conditions such as allergic lung inflammation, autoimmune diseases, transplant rejection and tolerance.^1^, ^2^, ^3^, ^4^, ^5^, ^6^, ^7^, ^8^ Here, we found the recently discovered cytokine IL‐38 to be a potent immune regulator that improved host immune reaction of CD4^+^CD25^+^Tregs together with prevention the development of sepsis. Our results showed that IL‐38 and its receptor were expressed in murine CD4^+^CD25^+^Tregs and that LPS stimulation up‐regulated IL‐38 expression. IL‐38 could enhance the immune response of CD4^+^CD25^+^Tregs, thereby amplifying Th2 polarization of effector T cells in sepsis. Furthermore, treatment with IL‐38 obviously decreased the mortality rate of CLP‐induced sepsis in mice, while blocking IL‐38 reduced the survivals of septic mice, thus identifying IL‐38 as a potential therapeutic agent against septic complications. In addition, our data confirmed that CD4^+^CD25^+^Tregs appeared to be required for the protection against sepsis elicited by IL‐38.

IL‐38, the most recently characterized member of the IL‐1 family cytokines, is abundantly expressed in various tissues and cells following stimulation with rhinovirus, inflammatory cytokines, bacteria, Toll‐like receptor (TLR) agonists or other pathogens, although IL‐38 expression in CD4^+^CD25^+^Tregs has been unclear.^31^, ^32^, ^33^ There is growing interest in investigating the regulatory pathway of IL‐38 in the pathogenesis of inflammatory and autoimmune disorders. High concentrations of IL‐38 have been observed in patients with asthma, chronic hepatitis B, rheumatic arthritis and Crohn's disease, and its expression is associated with the severity of these diseases.^34^, ^35^, ^36^, ^37^, ^38^, ^39^, ^40^, ^41^, ^42^ IL‐38 production is elevated in clinical sepsis and negatively associated with the number of blood leucocytes and expression of inflammatory cytokines.^43^ Moreover, rmIL‐38 administration can modulate inflammatory response and facilitate bacterial clearance.

Nevertheless, the potential role and underlying mechanism with regard to IL‐38 in the host immunity in the setting of sepsis remains to be elucidated.^43^ In the present study, our findings indicated that both IL‐38 mRNA and protein were expressed in CD4^+^CD25^+^Tregs and were strongly up‐regulated after LPS stimulation. Previous work revealed that LPS could activate CD4^+^CD25^+^Tregs via TLR4 signalling. Intriguingly, we noticed that IL‐38 could markedly improve the immune function of CD4^+^CD25^+^Tregs in sepsis. As we known, Tregs use different pathways to limit inflammatory and immune responses. In particular, Foxp3 and CD152 are recognized as critical markers that can determine the development and biological function of Tregs. These regulators are required for Treg‐induced immune suppression and homeostasis: deficiency of Foxp3 and CD152 impairs the immune activities of Tregs, causing fatal T cell‐induced autoimmune disease, systemic lymphoproliferation, and increased tumour immunity.^1^, ^2^, ^3^, ^4^ Herein, we found that prominent up‐regulation of Foxp3 and CD152 expression occurred in CD4^+^CD25^+^Tregs stimulated with IL‐38, and IL‐38 induced CD4^+^CD25^+^Tregs to augment the release of anti‐inflammatory IL‐10 and TGF‐β1. We also confirmed that these anti‐inflammatory cytokines help mediate the immunosuppressive activity of CD4^+^CD25^+^Tregs. IL‐10 and TGF‐β1 have been reported to down‐regulate immune response in animal models of various diseases including inflammatory bowel disease, colitis, tumours and diabetes.^1^, ^4^, ^5^, ^10^

It is established that helper T cells are classified into Th1 and Th2 subsets by cytokine production following stimulation, and Th1/Th2 imbalance contributes in the immune dysfunction.^19^, ^20^, ^21^, ^22^ Sepsis is typically characterized by initial hyper‐inflammatory Th1 response, which is associated with disease severity and mortality. Strikingly, Tregs are effective to maintain Th1/Th2 balance and dampen excessive pro‐inflammatory response and immunopathology during early‐phase of sepsis.^1^, ^3^ In the current study, we evaluated Treg‐mediated Th1/Th2 balance and associated cytokines including IL‐2, IL‐4 and IFN‐γ. After co‐culture with IL‐38‐stimulated CD4^+^CD25^+^Tregs, effector T cells switched towards Th2 response, as reflected by an increased ratio of IL‐4/IFN‐γ and elevated IL‐4, and reduced IL‐2 and IFN‐γ levels. In parallel, proliferation of effector T cells was greatly diminished. All these effects of IL‐38 were abolished by administration of anti–IL‐38 antibody, therefore providing evidence that IL‐38 could improve the immune activities of CD4^+^CD25^+^Tregs in sepsis.

IL‐38 may help counteract various inflammatory pathologies, as reflected by a decrease in local and systemic inflammation (eg Th17 cytokines, TNF‐α, IFN‐γ and chemokines), inhibition of receptor activator of nuclear factor kappa‐B ligand (RANKL) and improvement of bacterial clearance.^31^, ^32^ However, IL‐38 does not appear to affect intrinsic antibacterial activity of phagocytes.^43^ The present study showed that early replenishment of IL‐38 was associated with a higher survival rate in mice challenged with polymicrobial sepsis, indicating that IL‐38–mediated responses in CD4^+^CD25^+^Tregs could restrain early hyper‐inflammation and protect against septic complications. More importantly, IL‐38 appears to exert these benefits, at least in part, by regulating immune function of CD4^+^CD25^+^Tregs, as depletion of these cells decreases the survivals of septic mice treated with IL‐38.

The current study presents several limitations. Firstly, we verified the potential impact of IL‐38 by blocking IL‐38 activity with anti–IL‐38 antibody, but the effects of IL‐38 knockdown should be investigated in septic animal models. Secondly, instead of using an anti‐CD25 antibody, the role of conditional Foxp3 knockdown should be examined in a sepsis model under IL‐38 administration. Thirdly, our data with murine CD4^+^CD25^+^Tregs need to be explored in human Tregs, and ultimately, the clinical significance of IL‐38 should be elucidated in the clinical context.

The present study is to explore the effects of IL‐38 on CD4^+^CD25^+^Tregs and its beneficial action in sepsis. Future work is warranted to investigate the influence of IL‐38 on the signalling transduction involved in CD4^+^CD25^+^Tregs. Combination of IL‐38 with other cytokines may be a promising anti‐sepsis therapy.

## 5 CONCLUSIONS

Our results demonstrate enhanced expression of IL‐38 in CD4^+^CD25^+^Tregs in a murine sepsis model and highlight the immune activity of CD4^+^CD25^+^Tregs. The effects of IL‐38 can be reversed by anti–IL‐38 antibody. Strikingly, our data from CLP mice suggest that treatment with IL‐38 can notably improve the survival rate of septic mice and confirm that CD4^+^CD25^+^Tregs are required for the beneficial effects of IL‐38 in the development of sepsis.

## CONFLICT OF INTEREST

The authors declare no conflicts of interest.

## AUTHOR CONTRIBUTIONS

Y‐MY and YG designed the study. YG, MH, YW and ND performed the experiments and analysed the results. YG wrote the first draft of the paper, which Y‐MY revised. All authors read and approved the final manuscript.

## Data Availability

The authors declare that all relevant data supporting the findings of this study are available within the paper. Please contact Yong‐ming Yao (lead contact, c_ff@sina.com) for any enquiries.
